# An umbrella review on how digital health intervention co-design is conducted and described

**DOI:** 10.1038/s41746-024-01385-1

**Published:** 2024-12-23

**Authors:** Alicia Kilfoy, Ting-Chen Chloe Hsu, Charlotte Stockton-Powdrell, Pauline Whelan, Charlene H. Chu, Lindsay Jibb

**Affiliations:** 1 Lawrence Bloomberg Faculty of Nursing, University of Toronto, 155 College St, Toronto, ON M5T 1P8 Canada; 2 Division of Hematology/Oncology, The Hospital for Sick Children, 170 Elizabeth St, Toronto, ON M5G 1E8 Canada; 3 Child Health Evaluative Sciences, Peter Gilgan Centre for Research and Learning, The Hospital for Sick Children, 686 Bay St, Toronto, ON M5G 0A4 Canada; 4 Centre for Musculoskeletal Research, University of Manchester, Oxford Road, Manchester, M13 9PT UK; 5 Centre for Health Informatics, Division of Imaging, Informatics and Data Sciences, University of Manchester, Oxford Rd, Manchester, M13 9PL UK; 6 KITE Research Institute, University Health Network, 550 University Avenue #12-165, Toronto, ON M5G 2A2 Canada

**Keywords:** Medical research, Culture

## Abstract

Co-design has been suggested to improve intervention effectiveness and sustainability. However, digital health intervention co-design is inconsistently reported. This umbrella review aims to synthesize what is known about co-design of digital health interventions. We searched five databases from inception. Reviews which reported on co-design methodologies used in digital health were eligible. Information on review type, health conditions, and reported specifics of co-design were extracted and synthesized. Methodological quality was assessed using the AMSTAR2 tool. We included 21 reviews published between 2015 and 2023. Co-design participants included patients, caregivers and healthcare professionals. The frequency and breadth of participant involvement in co-design activities were reported in less than half of reviews. Participants evaluated intervention co-design as a positive process. All reviews were rated as critically low quality. This umbrella review highlights the inconsistent reporting of co-design in digital health. Here, we emphasize the importance of creating guidelines to direct co-design activities.

## Introduction

Digital health interventions are health interventions delivered through digital tools or communication technologies which collect, store, share and analyze health information for purposes of improving patient health and health care delivery^1^. This umbrella term includes health interventions delivered through a broad range of digital tools including but not limited to wearable devices, mobile apps, texting through smartphones, and telehealth^1–3^. The number and popularity of digital health interventions has rapidly grown over the past decade as part of increased interest in healthcare digitalization and its potential to improve access to (personalized) care at lower costs^3,4^. Digital health interventions have been associated with overall positive changes in disease self-management, clinical outcomes, and quality of life in patient populations including young adults, pediatrics, and older adults^5–7^. In addition, many studies have reported these interventions to be highly acceptable to patients, family members, and clinicians^8,9^. Clinically implemented digital health interventions can provide actionable data to support policy making and fiscally responsible allocations of public funding. Digital health data generated in a structured way also may provide academic and industry partners with evidence related to real-world innovation utility. Despite these successes, there is increasing awareness of field-related challenges including the rarity in which these interventions are subject to rigorous scientific evaluation, lack of adoption by end-users, and poor integration into routine clinical care^3^.

Design and development of digital health interventions is complex and the lack of end-user involvement in the process is a key contributor to limited intervention use by end-users—adversely effecting potential impact on health outcomes and sustained practice integration^10–12^. Co-design, a collective creative approach where varied stakeholders including patients, clinicians and policy makers are actively involved in the development, design and implementation of interventions, has been suggested to address this pitfall^13^. Co-design enables researchers and designers to embed the specific needs, attitudes, and values of the end users and key contributors early in intervention development while simultaneously pre-emptively identifying and addressing potential barriers to adoption^10–13^. Co-design also addresses, in part, the ethical imperative to engage patients, clients, and families meaningfully in research related to them.

Despite these potential positive impacts, co-design strategies are inconsistently implemented in digital health intervention development and often involve only limited end-user involvement, particularly by vulnerable populations^14^. Further, a clear understanding of how best to conduct co-design of digital health interventions from a practical point of view, especially when considering how to engage the diversity of health system users, remains elusive. This knowledge is critical for key audiences including health funders, policy makers and practitioners to know which co-design approaches and activities may be most useful to the development of effective interventions. This review aims to address this gap by answering the question: what is known about the practical methods to conduct co-design of digital health interventions (including setting, population, intervention details, session length, co-design strategy) and the breadth and depth of end-user involvement in the process?

## Results

**Fig. 1:**
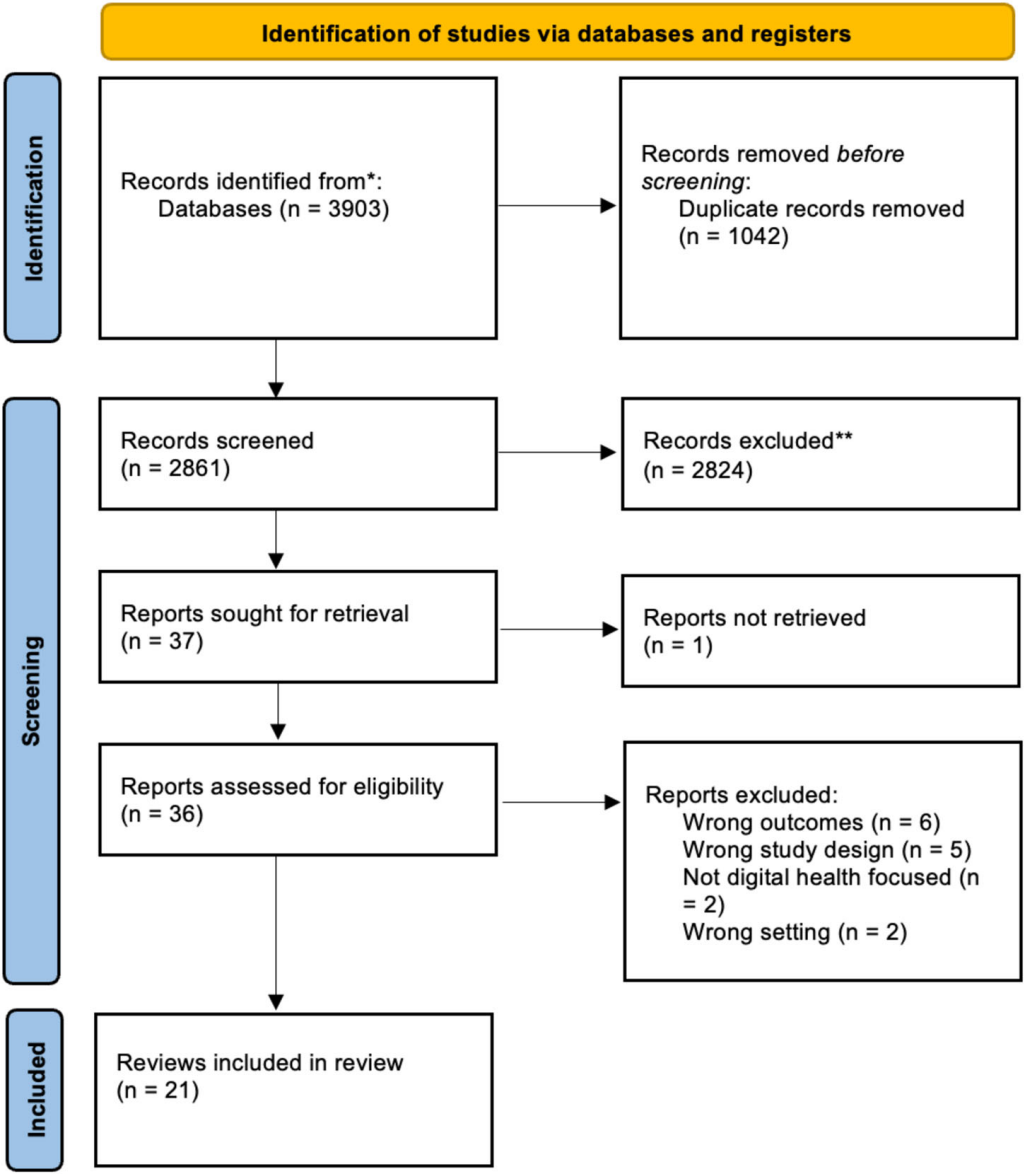
PRISMA flowchart article screening process. This flowchart, adapted from the PRISMA 2020 Flow Diagram, shows the number of records identified from the search (2861 non-duplicative records), the number of records excluded based on title and abstract (2824, and the number of studies excluded based on the full article review (15), and the reason for exclusions. Twenty-one reviews were included in this analysis.

Our search identified 3903 titles and abstracts. After excluding duplicates, we screened 2861 for inclusion and then 37 full-text articles. Twenty-one were included in the final analysis (Fig. 1). Reasons for exclusion included outcomes not matching inclusion criteria (*n* = 6), wrong study design (*n* = 5), not digital health focused (*n* = 2) and wrong setting (*n* = 2).

### Study characteristics

**Fig. 2:**
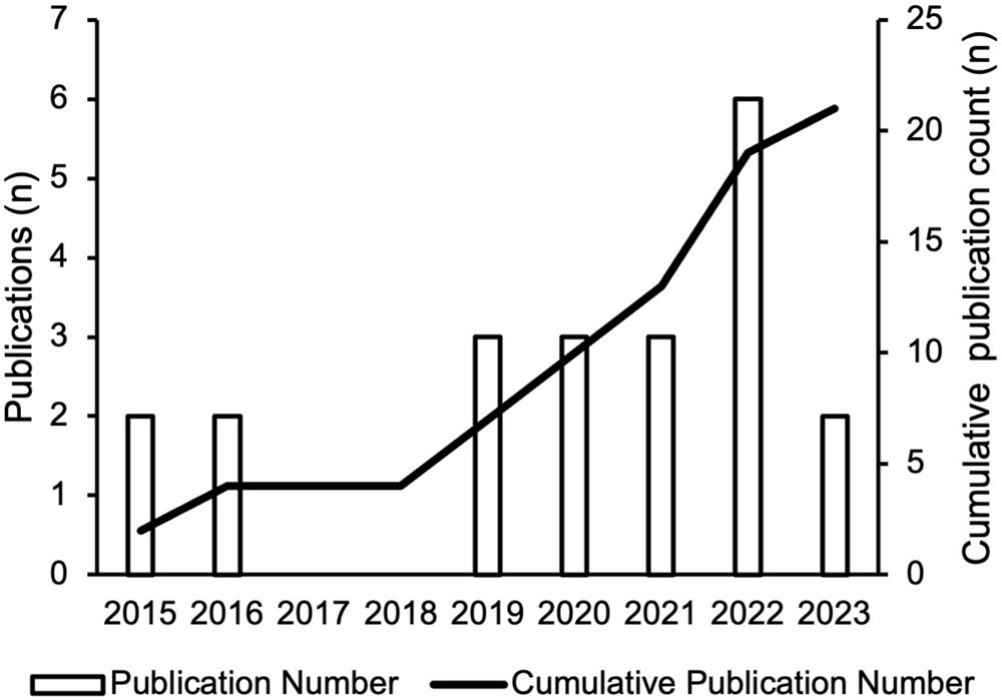
Number of reviews per year. This bar graph shows the number of studies (*y*-axis) published per year (*x*-axis). The cumulative number of reviews is also depicted by the line through the bar graph.

Table 1 presents the characteristics of included reviews. Reviews were published between 2015 and 2023, most often in Australia (*n* = 4)^15–18^, United Kingdom (*n* = 2)^19,20^, Canada (*n* = 2)^21,22^, United States (*n* = 2)^23,24^, the Netherlands (*n* = 2)^25,26^ and Denmark (*n* = 2)^27,28^. As shown in Fig. 2, papers were recent, most often published in 2022 (*n* = 6)^19,23,25,29–31^, 2021 (*n* = 3)^17,32,33^, 2020 (*n* = 3)^26,27,20^, 2019 (*n* = 3)^18,21,24^. Most reviews identified as systematic reviews (*n* = 11)^16,17,19,23,24,26,30–32,34,35^ or scoping reviews (*n* = 6)^18,21,25,27,29,28^. Other review types include literature reviews (*n* = 2)^15,32^, rapid review (*n* = 1)^22^, and practitioner review (*n* = 1)^20^. Reviews identified between 9^35^ and 433 studies^19^. Authors referred to interventions in their reviews as digital health (*n* = 6)^19,21,28,31,32,20^, mobile health (*n* = 6)^17,18,22,30,31,35^ or electronic health (*n* = 3)^23,25,26^. Other classifications included serious digital gaming interventions (*n* = 1)^34^, information communication technologies (*n* = 1)^24^, assistive technology (*n* = 1)^27^, technology-based interventions (*n* = 1)^16^, health-related technology (*n* = 1)^33^ and a combination of digital and mobile health (*n* = 1)^31^.

**Table 1:** Study, participant, and intervention characteristics

Primary author and publication year	Country	Databases and search range	Review type and number of studies	Intervention classification	Health condition	Population and age range	Co-design term(s) used	Goal of review
Baines et al.^19^	United Kingdom	Medline, Embase, PsycINFO, CINAHL, Scopus, ACM digital and gray literature (patient experience library database and google scholar)	Systematic review (k = 433)	Digital health interventions	Acute and chronic conditions (cancer, mental health, diabetes, breastfeeding, aphasia, human immunodeficiency virus, sexually transmitted disease, sleep, hearing loss and impairment) and health promotion	Age specifics not provided	Codesign, patient and public involvement, user-centered design, participatory design, cocreation.	Explore how patients and the public are involved in digital health innovation and to identify factors that support and inhibit meaningful patient and public involvement (PPI) in digital health innovation, implementation, and evaluation.
Baysari et al.^15^	Australia	Medline and Embase (2013–2014)	Literature review (k = 34)	Mobile health interventions	Chronic health conditions (e.g., diabetes, asthma, cancer), health promotion (breastfeeding)	Children to elderly patients (age range not provided)	Human factor approach	To examine what human factors methods, if any, were applied to the design, development, and evaluation oft the identified mobile applications.
Bevan Jones et al.^20^	United Kingdom	Medline, PsycInfo and Web of Science (inception to 2019)	Practitioner review (k = 25)	Digital health interventions	Chronic health conditions (depression, anxiety, sleep, self-harm, and suicide)	Children and young people (up to 18 years)	Co-design/development/production	To understand the development of digital mental health technologies in collaboration with CYP and other stakeholders.
Bird et al.^21^	Canada	Medline, CINAHL, Embase (inception to 2018)	Scoping review (k = 38)	Digital health interventions	Chronic health conditions (hematology/oncology/palliative, asthma, congenital heart disease, medical complexities, autism spectrum disorder)	Children (range not provided)	Human-centered design, end-user involvement, co-design	Describe the various models of synchronous home digital health that have been used in pediatric populations with special health care needs, their outcomes, and implementation barriers.
Cole et al.^23^	United States	PubMed, Embase and Scopus (2010–2021)	Systematic review (k = 25)	Electronic health interventions	Chronic health conditions (heart failure, mild cognitive impairment, hearing impairment, hypertension, diabetes, heart disease, asthma, prostatitis, hypotension, larynx cancer, COPD, reduced healing, eyesight, mobility, sensibility, loss of memory function) and health promotion in older adults	Older adults (at least 60 years of age).	Co-design, collaborative approaches, participatory design	Synthesize the current state of codesign approaches used to involve older adults in the development of EHTs.
Cwintal et al.^22^	Canada	Medline, Embase, Cochrane, Web of Science	Rapid review (k = 27)	Mobile health interventions	Chronic (oncology, COPD, spinal cord injury) and acute conditions (post-operative pain)	Child to adults (age range not provided)	Co-design	Summarize previously published uses of co-design in mHealth applications.
DeSmet et al.^34^	Belgium	PubMed, Web of Science, CINAHL, PsycInfo (2013–2014)	Meta analysis (k = 36)	Serious digital games	Healthy lifestyle promotion aiming to improve health behaviors, such as healthy diet, physical activity, social behavior, health responsibility and maintenance and stress management or self-actualization	Children-elderly (age range not provided)	Participatorydesign	Advance our understanding of how PD relates to game effectiveness by quantifying and comparing differences across studies and by overcoming small sample sizes in individual studies.
Eyles et al.^35^	New Zealand	Medline, EMBASE, PsycINFO, Scopus, CINAHL plus and google scholar (January 2005–January 2016)	Systematic review (k = 9)	Mobile health interventions	Chronic health conditions (schizophrenia, type 1 diabetes, mental health, traumatic brain injury, dementia, adolescents health and nutrition, positive emotion and social expressiveness, obesity) health behaviors (nutrition and physical activity, positive communication, and weight loss)	Adolescents-adults (age reported in 7 of the studies included in the review: 12–70 years)	Community-based participatory research (CBPR)	To identify and describe the methods and processes used for the co-design of mHealth interventions.
Henni et al.^29^	Norway	Medline, CINAHL, Scopus, IEE Explore, ACM library. Hand searched the journal of technology and persons with disabilities (2015–2020)	Scoping review (k = 25)	Digital health interventions	Chronic health conditions (cognitive, motor and hearing Impairments)	Children to adults (age range not provided)	Participatory and universal design	Investigate the needs and barriers of people with impairments related to use of digital health solutions and strategies to foster user participation, access, and utilization of digital health solutions.
Kip et al.^25^	Netherlands	Scopus, Google Scholar, and Web of Science (inception until 2021)	Scoping review (k = 160)	Electronic health interventions	Condition not reported	Children to older adults (age range not provided)	Human-centered development	To provide an overview of research activities used in studies guided by the CeHRes Roadmap.
Mitchell et al.^24^	United Sates	EBSCO, PubMed, and Web of Science (inception to 2017)	Systematic review (k = 57)	Information communication technologies	Healthy behaviors (weight management medication adherence and education, skin care, healthy behaviors for geriatric patients, postoperative health considerations and sexual health) and chronic diseases (cancer gout, lung disease, mental health, HIV, diabetes and kidney disease, arthritis, amblyopia, cardiovascular disease, lupus, autism, and chronic pain)	Children to older adults (5–78 years)	Patient-centered methods for design and development	Explore the current landscape of patient-centered design and development of health ICTs through a systematic review
Nimmanterdwong et al.^30^	Thailand	IEEE Xplore, PubMED/MEDLINE, Scopus (inception until November 2020)	Systematic review and narrative synthesis (k = 8)	Mobile health interventions	Chronic health conditions (heart failure, psychiatric disorders, fall risk assessment and detection, sarcopenia prevention, patients with cardiac implantable electronic devices)	Older adults (at least 60 years of age)	Human-centered design	Explore existing literature on relevant primary research and case studies to (1) illustrate how HCD can be used to create mHealth solutions for older adults and (2) summarize the overall process with recommendations specific to the older population
Nusir et al.^31^	Saudi Arabia	Web of Science, PubMed, Scopus and EBSCO-SocINDEX (2005–2020)	Systematic review (k = 22)	Digital and mobile interventions	Acute and chronic health conditions (COVID-19, psychological needs, diabetes)	Children to adults (age range not provided)	Co-design, collaborative design, participatory design, creative design, creative collaboration	To summarize how the co-design methodologies handled the existing technology-based health systems for their improvement
Øksnebjerg et al.^27^	Denmark	PubMed, PsycINFO, Web of Science, Scopus, Embase, and CINAHL. Hand search, Opengrey (inception to 2018)	Scoping review (k = 11)	Assistive technology	Chronic health condition (dementia)	Adults (age range not reported)	Involvement of end users in design and/or test phases	To explore and synthesize research addressing assistive technology designed to be used by people with dementia for self-management.
Orlowski et al.^16^	Australia	Medline, PubMed, PsycINFO, CINAHL, Scopus, Web of Science, Informit, arXiv.org, ACM Digital Library, and IEEE Xplore Digital Library (inception to June 2014)	Systematic review (k = 17)	Technology-based interventions	Chronic health conditions or well-being focus (autism, public mental health services, obesity, (mental illness and caregivers, sexual and mental health promotion for adult men who have sex with men, depression, behavioral issues, anorectal anomaly, online mindfulness therapy, self-harm, self-identified health concerns, alcohol use)	Youth only (10–26 years of age)	Community-based participatory research, participatory action research, participatory design, and user-centered design	To investigate consumer involvement processes and associated outcomes from studies using participatory methods in development of technology-based mental health and well-being interventions for youth
Sanz et al.^32^	Spain	PubMed (2017–2020)	Literature review (k = 20)	Digital health interventions	Chronic health conditions (cardiovascular disease, diabetes, RA, motor neurone disease, cancer, HIV, dementia, parkinson’s, diabetes, heart failure)	Older adults (age range not provided)	Co-design, co-creation, contribution	Identify the most implemented practices in health and social care service co-design for digital solutions to guide the co- design process in the ValueCare project; used to create or design a digital health solution or concept for patients and citizens
Sumner et al.^33^	Singapore	Medline, Embase, CINAHL, Web of Science, Scopus, OpenGrey and Business Source (2009–2019)	Systematic review (k = 43)	Health related technology	Chronic health conditions and health promotion in older adults (14 of the studies targeted specific medical conditions or problems such as cognitive or physical impairments)	Older adults (at least 60 years of age).	Co-design	To evaluate the effects and experiences of co-designed technology that support older adults to age in place
The University of Newcastle et al.^17^	Australia	ACM, Scopus, Web of Science (inception to 2019)	Systematic review (k = 61)	Mobile health interventions	Chronic health conditions (heart disease, diabetes, asthma, home-based health care, bipolar disorder, osteoarthritis, cancer, depression, HIV, schizophrenia, stroke). Health promotion (physical activity, mental health, nutrition, smoking cessation, menopause self-care, positive psychology, STI and drug usage)	Adolescents to elderly patients (age range not provided)	Co-design	Understand the scope of empirical mHealth studies that have used co-design in terms of (1) the targeted disease management and/or health promotion context, (2) the involved stakeholder groups, and (3) the methods they used in the different co-design phases
Vandekerckhove et al.^26^	Netherlands	Embase, Medline ALL, Web of Science Core Collection, CINAHL (Inception to 2019)	Systematic review (k = 69)	Electronic health interventions	Chronic health conditions (mental health was most frequently addressed) and health promotion	Not reported	Generativearticipatory design	Aimed to explore the reporting and substantiation of generative PD methodologies in empirical eHealth studies published in scientific journals to further develop PD methodology in the field of eHealth
Wegener et al.^28^	Denmark	PubMed, Scopus, Embase, and IEEE (2009–2020)	Scoping review (k = 22)	Digital health interventions	Chronic health condition (cognitive decline, cognitive dysfunction, neurocognitive impairment, motor dysfunction, frailty, vulnerability)	Older adults (at least 65 years of age)	Co-creation, user involvement	Aimed to explore how older people with frailty and impairment are involved in various parts of the design processes of digital health technologies and identify gaps or neglected steps in a user-involving design process
Woods et al.^18^	Australia	CINAHL, PubMed, PsycINFO, and EMBASE (2010–2017)	Scoping review (k = 21)	Mobile health interventions	Chronic health conditions (respiratory conditions, cardiovascular diseases, diabetes, and cancer)	Community-dwelling older adults (range not provided)	Patient-centered, user-centered, participatory, or user-centered design principles	Identify, summarize, and report on the development of consumer mHealth interventions for chronic condition self-management in the adult community-dwelling population in primary peer-reviewed studies

Most commonly, reviews focused on co-design of digital intervention targeted towards individuals with chronic conditions (*n* = 8)^18,29,21,27,28,30,32,20^ and a combination of individuals with chronic conditions and health promotion in healthy individuals (*n* = 8)^15–17,23,24,26,33,35^. Further, one review identified studies focused broadly on acute and chronic conditions and health promotion in healthy individuals (*n* = 1)^19^, health promotion in healthy individuals (*n* = 1)^34^ and a combination of acute and chronic conditions (*n* = 2)^22,31^. Finally, one review did not report on the specific conditions included^25^.

A variety of terms were used to refer to co-design including co-design (*n* = 3)^17,22,33^, human factor approaches (*n* = 1)^15^, participatory design (*n* = 3)^26,29,34^, community-based participatory research (*n* = 1)^35^, human-centered development (*n* = 2)^25,30^, patient-centered methods for design and development (*n* = 1)^24^, involvement of end-users in design and/or test phases (*n* = 1)^27^. The remaining studies used multiple terms to describe co-design throughout their reviews (*n* = 9)^16,18,19,21,23,28,31,32,20^.

Reviews most frequently (*n* = 17)^15–18,22–28,30,31–33,20,35^ aimed to summarize or synthesize the current state of co-design approaches used in their identified interventions and health conditions. In addition to synthesizing the current state of co-design activities, four reviews provided more specific goals including the study by Henni et al.^29^, who aimed to investigate the needs and perceived barriers of people with impairments as they pertained to user engagement with digital health interventions^29^.

#### Co-design participants

Most reviews (*n* = 19) included a range of co-design participants such as patients, caregivers, healthcare professionals, policy makers, teachers, and behavior specialists. The remaining two reviews^24,33^ reported on studies which only involved patients in co-design processes. Ten of 21^18,29,23,26–28,30,32,34,35^ reviews discussed co-design session sample sizes and, when reported by included studies, these ranged from 2^32^ to 1000^35^ participants.

Reviews reported on engaging patients ranging from children to older adults (*n* = 7 )^15,29,22,24,25,31,34^, older adults (≥60 years) (*n* = 6)^18,23,28,30,32,33^, adolescents to adults (*n* = 2)^17,35^, children and young people (*n* = 2)^16,20^, children (≤18 years) (*n* = 1)^21^, adults (*n* = 1)^27^, or did not provide data on participant ages (*n* = 2)^19,26^. Specific participant age ranges or definitions of what was meant by terms such as ‘children’ were often not reported. A single review presented data on study participant race and ethnicity^35^ and three reported on gender or sex^16,29,35^. The review by Eyles et al., which reported on race and ethnicity and gender or sex emphasized that in their identified studies information on age, gender and socioeconomic position of participants and stakeholders was generally poorly reported^35^.

### Co-design activities

All reviews identified described the co-design activities used by included studies, with wide variation in the level of detail provided. Surveys were the most frequently used quantitative approach to enable co-design; reported in 11 reviews^15,17,21,23–25,28,32,20,34,35^. Focus groups and interviews were also frequent; reported in 17^15,17–19,29,22–25,27,28,30,20,32,33,34,35^ and 14^15,17,19,29,22–25,27,28,30,20,32,34^ of reviews respectively. Other qualitative approaches reported in the reviews were observation (*n* = 10)^15,17,21,23,27,28,30,31,33,35^ and think-aloud strategies (*n* = 6)^18,19,23,31,33,20^. Various creative co-design activities were also reported, including storyboarding (*n* = 6)^16,17,26,30,20,35^, persona/scenario building (*n* = 6)^16,17,25,26,30,31^, drawing (*n* = 2)^33,20^, photos/video elicitation (*n* = 4)^18,29,33,35^, storytelling (*n* = 2)^31,20^, and role-playing (*n* = 1)^26^. Where digital prototypes were included in co-design, they were most frequently 2D or paper-based models (*n* = 6)^15,17,18,26,30,33^, wireframes (*n* = 3)^17,30,20^, or web-based software (*n* = 2)^33,20^. Reported intervention evaluations were iterative usability testing (*n* = 8)^15,17,18,29,22,25,28,30^, digital health intervention-embedded engagement metrics including app-tracking (*n* = 2)^15,20^, pilot testing (*n* = 4)^17,27,30,33^, and living laboratories (*n* = 3)^15,27,33^.

The locations where co-design activities were conducted was discussed in seven reviews^18,23,27,30,32,20,35^. Of reviews reporting on co-design location, one found that of the 25 studies identified, only 11 reported a specific setting. These settings were laboratories, clinics, homes, community, senior centers, and virtual; however, the specific number of studies reporting each location was not provided^23^. The remaining reviews provided scant detail on locations.

### Reporting on co-design frequency, duration and degree of participation

The duration and frequency of co-design sessions was reported by less than half of the reviews; with only eight^18,24,27,30,32–34,35^ discussing session duration and seven^18,27,28,32,33,34,35^ reporting session frequency. Within those studies, frequency and duration were reported in varying detail. Most reviews reported a handful of examples from the studies they identified, including a 2-h collaborative design workshop or a half-day co-design workshop^33^. Reviews by Eyles et al.^35^ and Woods et al.^18^ reported that the studies they identified provided inadequate descriptions of both session duration and frequency.

Seventeen of the studies^15–17,19,21–28,30,31,20,33,35^ made attempts to distinguish during which part of intervention pre-design, development, evaluation and post-design participants were included in. The review by Cole et al.^23^ rated level of co-design participation using a framework^36^ with ratings being “informed”, “consulted”, “involved”, “in collaboration as a co-leader” and “empowering oneself and others”. Of the 25 studies, Cole et al. states that most involved the first three levels of participation. The review by Orlowski et al.^16^ also made attempt to classify participant involvement in co-design through categorizing studies using several different concepts drawn from participatory based research^16^. Overall, they found that 70% of projects reported predominantly consultative consumer involvement^16^.

Twelve of the reviews^15,17,18,21,24–26,28,30,32,20,35^ provided details on the aspects of the intervention that end-users participated in co-designing, although to varying degrees. For example, the review by Bevan Jones et al. highlighted a study in which discussions with youth patient partners focused on illustrations, characters, scripts and animation for the digital health intervention^20^. The study by Wegener et al. provided a detailed list of specific contributions older participants provided to identified intervention development including content of applications and how censors should be worn^28^.

### Frameworks used to guide co-design and review conduct

Thirteen reviews^16–18,21–26,28,30,31,35^ aimed to identify frameworks or theories which underpinned intervention development including behavioral change, intervention development and co-design frameworks. Of these thirteen reviews, all except one identified frameworks used in studies^21^. Thirty-two frameworks, models or theories related to co-design were described by reviews. The most used were variations of the participatory design (PD) method, user-centered design and human-centered design frameworks. Authors of reviews also used co-design frameworks or models to synthesize results, with ten of 21^17,18,21,23,25,26,32–34^ reporting such use.

### Evaluation of co-design

Co-design was evaluated in terms of (1) overall effectiveness and (2) participants’ views of the process. Reviews reported that quantitative evaluation of co-design effectiveness was overall challenging and only one review provided a meta-analysis^34^. This meta-analysis did not support the notion that digital games developed with participatory design improve health outcomes more than those not co-designed^34^. The review by Vandekerckhove et al.^26^ reported a series of outcomes including eHealth development (number of ideas for development), eHealth quality (usability, feasibility) and user outcome (effectiveness) which were reported in their identified studies^26^. Qualitative reports on the potential for co-design to improve digital health intervention utility were reported by three reviews^19,21,24^. These reviews stated that an end-user advisory group can lend valuable insights into intervention content and structure, making interventions more user-friendly and feasible to implement^21,24^ and that adoption of participatory approaches to the design of eHealth interventions and the use of personalized content enhances overall system effectiveness^19^. Five reviews^16,22,26,32,20^ reported on participants’ views on participating in co-design and overall reported high levels of satisfaction; however, most of these reviews emphasized that this was an infrequently assessed quality metric in identified studies.

### Co-design barriers and challenges

Nine reviews^16,18,19,22,25,28,30,33,20^ reported on challenges to co-design of digital health interventions. Power imbalances between researchers and participants were amongst the most cited barriers to co-design conduct. Additional barriers included time and financial constraints, costs, difficulty recruiting participants particularly participants from a minority or vulnerable group, participant “groupthink” at co-design sessions, and the thoughts of medical and health professionals being privileged than that of patients. Two reviews reported barriers to specific co-design strategies^25,20^ which included perceived inadequacies of surveys and questionnaires in exploring complex issues as well as difficulties participants faced in freely talking to strangers in new settings, including in focus groups^20^. The review by Sumner et al.^33^ listed barriers to successful co-design of digital health interventions and proposed subsequent strategies to address them. These strategies included building relationships and trust, empowering the end-user, building end-user knowledge, and establishing value and interest^33^. It was suggested that lacking buy-in from researchers and participants, as well as issues with recruitment, could be addressed through conducting co-design in environments familiar to participants^33^.

### Accessibility and equity

Eight reviews reported on accessibility and equity^29,21,22,26,28,30,20,35^. Bevan Jones et al. identified a study which discussed the inclusion of cultural advisors and hosting formal design opening and closing sessions with community elders in the Maori and Pacific Islander populations^20^. Identified strategies to recruit vulnerable populations discussed in reviews included using a proactive outreach approach which involved using a combination of approaching and recruitment strategies. Other reviews highlighted that not embedding equity and accessibility principles in co-design of digital health interventions risked worsening the digital divide^21^ and design failures if developer biases and stereotypes related to certain groups, such as older adults, were embedded in products^30^. A handful of the reviews identified focused specifically on improving co-design in vulnerable populations including children with special health care needs and their families^21^, people with impairments^29^, people with dementia^27^ and older people with frailty or impairment^28^.

### Review quality appraisal

All twenty-one reviews were classified as critically low quality. Very few studies met the requirements for questions one (*n* = 2)^33,34^, seven (*n* = 2)^22,26^, nine (*n* = 1)^33^, 10 (*n* = 1)^16^, 11 (*n* = 1)^34^, and 13 (*n* = 1)^30^. No studies met the requirements for questions 12 and 15. For additional details and full questions see Table 2.

**Table 2:** AMSTAR-2 results

Source	1	2	3	4	5	6	7	8	9	10	11	12	13	14	15	16
Baines et al.^19^	No	P. Yes	No	P. Yes	Yes	No	No	No	No	No	NMA	NMA	No	No	NMA	Yes
Baysari et al.^15^	No	No	No	P. Yes	Yes	No	No	No	No	No	NMA	NMA	No	No	NMA	No
Bevan Jones et al.^20^	No	No	No	P. Yes	Yes	Yes	No	No	No	No	NMA	NMA	No	No	NMA	Yes
Bird et al.^21^	No	No	No	P. Yes	Yes	Yes	No	P. Yes	No	No	NMA	NMA	No	No	NMA	Yes
Cole et al.^23^	No	P. Yes	No	P. Yes	Yes	No	No	No	No	No	NMA	NMA	No	Yes	NMA	Yes
Cwintal et al.^22^	No	P. Yes	No	P. Yes	Yes	Yes	Yes	No	No	No	NMA	NMA	No	No	NMA	Yes
DeSmet et al.^34^	Yes	No	No	P. Yes	No	Yes	No	No	No	No	Yes	No	No	No	No	Yes
Eyles et al.^35^	No	P. Yes	No	P. Yes	No	No	No	Yes	No	No	NMA	NMA	No	No	NMA	Yes
Henni et al.^29^	No	No	Yes	P. Yes	No	No	No	No	No	No	NMA	NMA	No	Yes	NMA	Yes
Kip et al.^25^	No	No	No	P. Yes	Yes	No	No	No	No	No	NMA	NMA	No	No	NMA	Yes
Mitchell et al.^24^	No	No	No	P. Yes	Yes	Yes	No	No	No	No	NMA	NMA	No	No	NMA	Yes
Nimmanterdwong et al.^30^	No	No	Yes	P. Yes	Yes	No	No	No	No	No	NMA	NMA	Yes	Yes	NMA	Yes
Nusir et al.^31^	No	No	No	P. Yes	No	No	No	No	No	No	No	No	No	No	No	No
Øksnebjerg et al.^27^	No	No	No	P. Yes	Yes	No	No	No	No	No	NMA	NMA	No	Yes	NMA	Yes
Orlowski et al.^16^	No	No	No	P. Yes	No	No	No	No	No	Yes	NMA	NMA	No	No	NMA	Yes
Sanz et al.^32^	No	No	No	No	No	No	No	No	No	No	NMA	NMA	No	No	NMA	Yes
Sumner et al.^33^	Yes	P. Yes	Yes	P. Yes	No	Yes	No	P. Yes	Yes	No	NMA	NMA	No	Yes	NMA	Yes
The University of Newcastle et al.^17^	No	No	No	P. Yes	No	No	No	No	No	No	NMA	NMA	No	No	NMA	No
Vandekerckhove et al.^26^	No	No	Yes	P. Yes	Yes	No	P. Yes	No	No	No	NMA	NMA	No	No	NMA	Yes
Wegener et al.^28^	No	No	Yes	P. Yes	Yes	No	No	No	No	No	NMA	NMA	No	No	NMA	Yes
Woods et al.^18^	No	No	Yes	P. Yes	No	No	No	No	No	No	NMA	NMA	No	Yes	NMA	No

*P. Yes* partial yes, *NMA* no meta-analysis.

Question Legend

1. Did the research questions and inclusion criteria for the review include the components of PICO?

2. Did the report of the review contain an explicit statement that the review methods were established prior to the conduct of the review and did the report justify any significant deviations from the protocol?

3. Did the review authors explain their selection of the study designs for inclusion in the review?

4. Did the review authors use a comprehensive literature search strategy?

5. Did the review authors perform study selection in duplicate?

6. Did the review authors perform data extraction in duplicate?

7. Did the review authors provide a list of excluded studies and justify the exclusions?

8. Did the review authors describe the included studies in adequate detail?

9. Did the review authors use a satisfactory technique for assessing the risk of bias (RoB) in individual studies that were included in the review?

10. Did the review authors report on the sources of funding for the studies included in the review?

11. If meta-analysis was performed did the review authors use appropriate methods for statistical combination of results?

12. If meta-analysis was performed, did the review authors assess the potential impact of RoB in individual studies on the results of the meta-analysis or other evidence synthesis?

13. Did the review authors account for RoB in individual studies when interpreting/ discussing the results of the review?

14. Did the review authors provide a satisfactory explanation for, and discussion of, any heterogeneity observed in the results of the review?

15. If they performed quantitative synthesis did the review authors carry out an adequate investigation of publication bias (small study bias) and discuss its likely impact on the results of the review?

16. Did the review authors report any potential sources of conflict of interest, including any funding they received for conducting the review?

## Discussion

This umbrella review provides insight into what is known about the practical methods used in the co-design of digital health interventions, the breadth and depth of end-user involvement in the process, and the characteristics of included end-users. Overall, we highlighted the inconsistent and poor reporting of co-design activities used in digital health intervention design and in the reviews. Most reviews reported the inclusion of a broad range of co-design participants including patients, caregivers, healthcare professionals, policymakers, teachers, and behavior specialists; however, the demographic profile of participants known to be engaged in designing digital health interventions is inconsistently reported. All reviews reported on the co-design activities used in studies, including interviews and surveys, however very few described specifics of the sessions including underpinning methodological frameworks, frequency, intensity, and location. Evaluation of co-design effectiveness, in terms of impact on intervention functionality and participant views on participating in the process, were infrequently reported. Reported barriers to co-design included power imbalances and lack of buy-in by researchers, and relationship building and establishing participant value and interest were considered mitigating factors to such challenges. Accessibility and cultural sensitivity were discussed in less than half of the reviews but, when present, centered on recruiting diverse populations to improve representation, and the inclusion of cultural advisors to create more welcoming environments and cultural respect.

Reviews were most frequently published in recent years, a finding likely reflective of the major growth in the field of digital health and the growing appreciation for the need to involve end users in product design^37^. Increased interest in the principle of co-designing interventions has occurred across fields, including in artificial intelligence^38^, non-digital health interventions and educational interventions^39,40^, as involving end-users is considered to reduce biases^41^, increase engagement and intervention effectiveness^42^. Reviews were also only conducted in high-income countries. Given the broad potential for digital technologies and artificial intelligence to improve the access to and acceptability of healthcare, reporting on co-design processes from the perspectives of users, particularly those in including in low- and middle-income countries is required^43^.

Terminology used in reviews to describe the principle of co-design varied and included “patient and public involvement”, “user-centered design”, “co-creation” and “human factors approaches”. These terms were used interchangeably by authors and involved very similar methodologies. This likely reflects how the concept of end-user involvement in design and development has evolved over time^44–46^ and across fields. Standardizing the term used may minimize confusion, create a sense of cohesion across the disciplines of healthcare, engineering, and software developments, as well as ensure methodologies are implemented rigorously and consistently.

All reviews reported on the specific co-design activities used in their identified studies. Strategies were surveys, interviews, and focus-groups, among others; however, very little detail was provided about the practical methods employed, including the intensity and frequency of co-design sessions or when to best implement these strategies during the design, development, and evaluation processes. Further, although several reviews did endeavor to describe the degree of participant involvement in the design, development, and evaluation process, only two reviews qualified this using a participatory framework^16,23^. Such information is critical to an understanding of the breadth and depth of meaningful end-user participation in digital health intervention development and has been recognized as such in healthcare research more broadly^39,40^. Future research reports should aim to address these gaps through detailed description of the specific co-design activities and processes used. In addition, only approximately half of reviews reported on the specific features of the intervention which participants co-designed including technical requirements and content. Inclusion of this detailed information is critical in reviews to ensure future researchers can use the information as a guide for their own studies.

Reporting on the details of those participants involved in digital health co-design, including profiles of their age, sex, gender, race, ethnicity, socio-economic status, and health status were scant. Such reporting is required to provide evidence regarding health impacts, satisfaction with care, disparities and inequities experienced across demographic groups and is recommended as best practice in research^47–49^. Historically, design work has engaged so-called “super-users” (users who frequently contribute to research projects) due to their comfortability with the research and ability to contribute^50,51^. Such participants are often not representative of the population for whom a digital health intervention is designed, potentially exacerbating digital divides, and shrinking intervention effectiveness^43,52^ for groups such as older adults, young children, and those with low health or digital literacy^43,52,53^. Research is needed to understand how best to recruit such groups into co-design work, which co-design methods may be most appropriate for different groups, and how to support meaningful engagement throughout the process. One way this can be accomplished is through ensuring that locations and settings/environments of co-design sessions are accessible for participants, whether in their own community rather than an academic setting or through virtual means^43,52,53^.

Utilizing a framework or theory to underpin methodological choices is one way to increase quality research, including co-design methods^54^. As a form of participatory research, it is critical researchers maintain congruency between their epistemological, theoretical, and methodological decisions^55^ to increase the rigor of their co-design research. Despite this, only half of reviews either utilized a framework to guide their review or highlighted the frameworks identified in their studies. Framework-, model- or principles-based digital health co-design, which is underpinned by empirical evidence, should be a focus of future research.

Finally, our review indicates a need for improved reporting on the impact of co-design in terms of the effectiveness of generated digital health products and participant experiences. This concern could be reflective of inconsistent reporting at the study level. Previously identified as problematic within the co-design literature^56^, efforts have been made to develop robust tools and frameworks to evaluate patient and public engagement^56,57^. Rigorous and on-going evaluations of co-design activities are required to determine the degree of end-user engagement and learn how to improve practices in the future^56^. Participant involvement in intervention design and development also has potential to create a sense of empowerment by building knowledge bases and advocacy skills^58^.

Considering the quality of included reviews, all were scored using the AMSTAR 2 tool as critically low. AMSTAR 2 items where reviews scored particularly low included those related to description of included study designs. Reviews also provided sparse detail on their constituent studies, making it difficult to understand who was included in co-design, how co-design was conducted, and how it evaluated. We focused only on the data presented in reviews and therefore we cannot report on whether this lack of detail extends to reporting in the primary studies included in each review.

This review of review is not without its own limitations. We included a broad range of review types including scoping, systematic and narrative synthesis which challenged our ability to compare review findings. We also only included reviews published in English, potentially limiting understanding of these co-design understandings in other languages and cultural contexts.

Our umbrella review has synthesized data reported by reviews focused on co-designing digital health interventions and summarized what is known about co-design methods, the breadth and depth of end-user involvement and the characteristics of included end-users. Although we identified the co-design activities most frequently used, due to underreporting of information in reviews, we were limited in our ability to determine the details of these activities, including who participated. Few reviews reported on the evaluation of co-design activities and we found little consensus on the most appropriate framework or methodology to guide co-design. This reflects a lack of standardization and consistency across the field of digital health co-design. Currently available guidelines for patient and public involvement in intervention development include the Guidance for Reporting Involvement of Patients and the Public (GRIPP)^59^ and the GRIPP2^60^. However, these guidelines are generic and not specific to co-design in digital health^61^. Recommendations for governance and innovation in responsible digital health development highlight inclusive co-creation as best practice, with co-design capable of supporting digital healthcare that is clinically, ethically, and fiscally responsible^62,63^. Instruments to rate the quality of end-user involvement and associated reporting are required to create investigator accountability as it pertains to digital co-design.

In Table 3 we overlay the major findings of this review with practical suggestions for digital health co-design practitioners and scientists in the field. In doing so, we suggest efforts are needed to develop standardized guidelines for reporting co-design methodologies and to direct specific co-design methods and processes, with emphasis on guidance around the strategies that may be most engaging and effective in particular populations and health conditions. Ultimately, to be truly emblematic of co-design principles, these guidelines should be co-created with patients and caregivers and include meaningful involvement of healthcare professionals to enhance capacity to create clinically relevant health tools^64^. Such work has potential to ensure co-design as a principle in digital health development continues to evolve and leads to effective and sustainable interventions.

**Table 3:** Lessons and learned and practical suggestions for the development of digital co-design reporting guidance

Category of analysis	Central review finding	Practical suggestions for the development of digital co-design reporting guidance
Study characteristics	Reviews use varied terms to describe co-design and its activities, limiting capacity to identify, compare, and contrast literature.	• Overall, the co-design field of science, would benefit from standardized terminology for co-design concepts and related methodology that should be developed using consensus building between researchers and digital health end-users (e.g., through a Delphi survey).
Co-design participants	Co-design participants range greatly in characteristics including health condition and age; however little other sociodemographic participant data are typically presented.	• Determination of a set of minimally required participant sociodemographic data to contextualize findings should be co-created with end-users and implemented in each co-design project. • Recommended characteristics include number of participants engaged in co-design; participant type (e.g., patient, carer, healthcare professional, policy maker etc.); participant-identified age, sex, gender, race, ethnicity, education level, rurality, socioeconomic status, available digital infrastructure, material hardship, and health status.
Co-design activities	While the types of co-design activities used are reported details on activity specifics (e.g., location of activity) are underreported.	• End-users should be engaged in decisions related to the nature of activities used to co-design digital health prior to co-design sessions. • The process for engagement and details of activity specifics should be reported to contextualize findings and allow for reproducibility where appropriate.
Reporting on co-design frequency, duration and degree of participation	Reviews sporadically report on co-design session frequency, duration and the degree of end-user participation in co-design activities.	• Recommended reportable co-design activity details include the frequency and duration of engagement, the specific aspects of intervention design that included end-users, and level of end-user co-design participation and decision-making. • Establish a transparent process for documenting ideas that are not integrated into the final intervention, including providing feedback to participants on why certain suggestions were not used and storing these ideas for potential use in future projects. Recognizing such contributions fosters respect, encourages continued engagement, and helps build a repository of ideas that might inform future design cycles or related projects.
Frameworks used to guide co-design and review conduct	Several theoretical and methodological frameworks were used to underpin co-design activities, without explanation for framework selection.	• Framework-informed co-design research should detail framework specifics and the rationale for framework selection.
Evaluation of co-design	The effectiveness of co-design activities (i.e., the development of useful or effective digital interventions) and co-design participant satisfaction with the process are inconsistently measured or reported.	• Establish clear success metrics aligned with project goals (e.g., usability, satisfaction) to ensure co-design evaluations are comprehensive and meaningful. • An evaluation plan for co-design activities should be developed a priori and should consider participant satisfaction with the co-design process. • Both qualitative and quantitative measures of evaluation should be considered and results from the plan should be included in the reporting of the findings. • Incorporate reflection sessions following co-design to gather participants’ perspectives on the experience as part of the evaluation.
Co-design barriers and challenges	Reviews reported on barriers to co-design, including time and financial constraints, as well as challenges to the process, including recruitment and participant groupthink.	• Potential barriers and challenges, and related solutions, should be identified with end-users before co-design activities occur. • The process and duration of identification and solution building should be reported, as should any unforeseen challenges arising during co-design and methods used to mitigate these.
Accessibility and equity	Considerations related to accessible and equitable co-design are infrequently reported, limiting understanding related to fair involvement in digital health innovation. Reviews suggested including cultural advisors, equitable recruitment strategies, and the creating environments that address varying comfort levels with digital health.	• Accessibility and equity issues within the population should be surveyed prior to implementing activities, addressed accordingly (e.g., accommodating varying access to technology, scheduling needs, and comfort levels with research), and reported. • Participants engaged in activities should be surveyed during the co-design process and after to understand and address equity concerns or improvement suggestions during co-design; this process should be reported. • Fair compensation should be provided to participants, which can increase accessibility and equity by decreasing financial barriers, particularly for those from underserved and underrepresented communities.

## Material and methods

### Study design, literature search and study selection

A systematic review of reviews was undertaken, which is the recommended approach in instances where the amount of research in an area is expected to be large^65^. Our reporting is in accordance for the reporting guideline for overviews of reviews of healthcare interventions: PRIOR statement (Supplementary Table 1)^66^. No ethics approval was needed due to the nature of the manuscript. With the assistance of three research librarians (one specialized in nursing research, one in engineering and one pediatric hospital librarian), we searched PubMed/Medline, Embase, PsycInfo, Cochrane Reviews and Association for Computing Machinery (ACM) Digital Library from inception to March 8, 2023. Our search strategy (Supplementary Table 2) was developed using combinations of key words for digital health, telemedicine, and co-design. Our search strategy was created following an initial consultation with a university-based librarian specializing in healthcare literature. A subsequent consultation meeting was held with a university-based librarian specializing in engineering, who adapted our search for the ACM Digital database. The protocol for this umbrella review was not registered, however a detailed protocol was prepared through group discussion and can be accessed at request.

Included reviews reported on co-design methodologies used in digital health interventions. We defined co-design as the active involvement of end-users in the design and development of digital health interventions^12–14^. Further, we also included studies which assessed end-user involvement in implementation and evaluation if digital health interventions.

We defined digital health interventions as the use of information and communication technologies in medicine and other health professions to manage illness and health risks or promote wellness^67^. All types of digital modalities were included. Categories which fall under digital health included, but were not limited to, telemedicine, electronic health, mobile health, virtual gaming, virtual reality, chat bots, remote monitoring, and wearable digital devices. We included reviews focused on co-design of an intervention aimed at managing an acute (sudden onset involving <3 months and a return to the patient’s baseline likely^68^) or chronic (lasting >3 months or occurring three times or more in 1-year and requiring ongoing medical attention or limiting activities of daily living^69^) health condition or promoting healthy lifestyle habits. To be included end-users of digital health interventions must have been patients or the public. However, co-design activities could involve a wide range of participants including patients, caregivers, policy makers and software engineers so long as patients or the public were involved in some capacity.

To be included, reviews could focus on co-design of the health intervention or digital tool itself. Reviews must have: addressed any of co-design methods, co-design setting, or degree of end-user involvement; been written in English; and searched one or more databases using a systematic approach to identify studies. Two authors (A.K. and TCC.H.) piloted our application of the inclusion and exclusion criteria using 25 randomly selected abstracts and decision agreement was 100%. All title and abstract, and then full-text, screening was conducted in duplicate (A.K. and TCC.H.) in Covidence. Conflicts were resolved by discussion and any remaining were resolved by an independent third reviewer (L.J.).

### Data extraction and data synthesis

Two authors (A.K. and TCC.H.) extracted study data into an author developed and piloted codebook. Agreement on data extraction was 90% and frequent process meetings were used to resolve disagreements and reach 100% agreement. Data abstraction fields were grouped according to key data features to enable synthesis. These fields include study characteristics (publication year, country of origin, type of review, digital health intervention, etc.), co-design participants, and co-design activities. In order to understand the breadth and depth of end-user involvement in co-design we extracted frequency (how often activities took place), duration of co-design activities, degree of end-user participation throughout the co-design or evaluation process and aspects of the intervention (e.g., clinical content, tool appearance, hardware function) that end-users participated in co-designing. Quantitative data were analyzed using descriptive statistics and presented narratively.

### Quality appraisal

The quality of each included review was assessed in duplicate (A.K. and TCC.H) using the A Measurement Tool to Assess Systematic Reviews 2 (AMSTAR-2)^70^. Rating discrepancies were discussed and resolved with the author group.

## Supplementary information

Supplemental Material

## Data Availability

This study is an umbrella review, and it does not generate any new data. Questions regarding data access should be addressed to the corresponding author.
